# Flight behaviour of honey bee *(Apis mellifera)* workers is altered by initial infections of the fungal parasite *Nosema apis*

**DOI:** 10.1038/srep36649

**Published:** 2016-11-09

**Authors:** Ryan Dosselli, Julia Grassl, Andrew Carson, Leigh W. Simmons, Boris Baer

**Affiliations:** 1 Centre for Integrative Bee Research (CIBER), ARC Centre of Excellence in Plant Energy Biology, Bayliss Building (M316), The University of Western Australia, Crawley WA 6009, Australia; 2 Centre for Evolutionary Biology, School of Animal Biology (M092), The University of Western Australia, Crawley WA 6009, Australia.

## Abstract

Honey bees (*Apis mellifera)* host a wide range of parasites, some being known contributors towards dramatic colony losses as reported over recent years. To counter parasitic threats, honey bees possess effective immune systems. Because immune responses are predicted to cause substantial physiological costs for infected individuals, they are expected to trade off with other life history traits that ultimately affect the performance and fitness of the entire colony. Here, we tested whether the initial onset of an infection negatively impacts the flight behaviour of honey bee workers, which is an energetically demanding behaviour and a key component of foraging activities. To do this, we infected workers with the widespread fungal pathogen *Nosema apis*, which is recognised and killed by the honey bee immune system. We compared their survival and flight behaviour with non-infected individuals from the same cohort and colony using radio frequency identification tags (RFID). We found that over a time frame of four days post infection, *Nosema* did not increase mortality but workers quickly altered their flight behaviour and performed more flights of shorter duration. We conclude that parasitic infections influence foraging activities, which could reduce foraging ranges of colonies and impact their ability to provide pollination services.

The lifestyle of social hymenopteran insects, as found in all ants and some bees and wasps, results in related individuals living in close proximity to each other inside the colony, which offers parasites highly favourable conditions to spread and multiply^1^. Social insects are indeed known to host a wide range of different parasites such as viruses^2^,^3^, bacteria^1^,^4^ fungi^5^,^6^, protozoa^7^,^8^, as well as arachnids^9^,^10^ or other insects^11^,^12^ that can pose substantial threats to these societies. In the case of the honey bee (*Apis mellifera*), their impact has been identified as a main contributing factor towards the massive losses as observed in some managed and wild honey bee populations^10^,^12^.

Social insects are not defenceless however, and their immune systems consist of several adaptations to detect and combat pathogens^1^,^13^ such as social immunity, which includes a number of behaviours that reduce the spread of pathogens including grooming of infected relatives, or hygienic behaviour to remove parasitised brood from the colony^13^,^14^,^15^. Parasitised honey bee workers are known to start foraging at a younger age to reduce the risk of spreading disease to their nestmates^16^,^17^,^18^. Social insects also possess individual-based innate immune systems, and the individual’s ability to combat parasites is of central importance for colony fitness^19^. These consist of a cellular response to combat large parasites (through processes such as encapsulation and melanisation), as well as a humoral response mediated by antimicrobial peptides, proteins and other cytotoxic compounds^20^,^21^,^22^. The activation and use of such defence mechanisms is complex^23^ and assumed to be costly and to trade-off with other life history traits^24^. For example, immune activation reduces the survival of infected workers in the bumblebee *Bombus terrestris*^25^ and affects reproduction in honey bees by diverting their energy stores towards immunity^26^,^27^. Trade-offs between immunity and other life history traits seem also present in queens of the leaf cutting ant *Atta colombica*, where the capacity to activate the immune system during colony foundation is inversely proportional to the number of sperm stored during matings^28^.

If parasitic infections spread to an increasing number of colony members and impact their task performance such as brood care, grooming or foraging, their effects eventually become visible at the colony level^1^,^20^. In the most extreme case, they can result in the collapse and death of the entire colony, a phenomenon that has been frequently reported in honey bees and therefore received growing interest over recent years^19^. However, how parasite-driven stress acting at the individual level eventually translates into the collapse of an entire colony remains to be studied in detail.

Here, we quantified the initial impact of an infection on cellular immune response and flight behaviour of a cohort of same-aged honey bee workers. Our experimental setup covered the time frame of establishment of a primary infection and the consequent physiological and behavioural responses of the host. We predicted that the response of the immune system to infection is costly, which bees compensate for by reducing the duration or frequency of their foraging trips. To do this we took workers with an age of 18–19 days, which coincides with the start of their foraging activities^29^ and infected them with the widespread fungal pathogen *Nosema apis*. Infections of this obligate parasite occur when spores are ingested and multiply in the epithelial cells of the mid gut. The pathogen can eventually be transmitted to other bees through faecal contaminations^30^,^31^ or during mating from males to queens^32^. *N. apis* infections are often phenotypically expressed by dysentery^30^,^33^,^34^ and increased hunger levels of workers^35^,^36^ resulting in elevated sugar consumption^37^,^38^. *N. apis* is typically referred to as a parasite with low virulence^10^,^37^, and parasite spores are indeed recognised and killed by the immune system of honey bee males^39^. Furthermore, infections result in a complex but very specific response of the innate immune system, but also induce substantial changes in the expression of core metabolic pathways that are similar to those observed during energetic stress^34^. In some cases, *N. apis* infections can spread and cause colony death, although this seems driven by the co-occurrence of additional stress factors such as unfavourable climatic conditions, pesticide exposure or the presence of additional infections with other pathogens^40^,^41^,^42^.

We used flight behaviour of worker bees as a response variable because it is energetically demanding, and metabolic activity of foragers has previously been found to be substantially higher compared to individuals that remain inside the colony^43^. Flight activity is also a key component of foraging, and therefore a determinant of colony performance and fitness, because food shortages negatively impact on immune defence or brood care in honey bees^44^,^45^,^46^,^47^. To monitor flight behaviour of individual bees in response to a *N. apis* infection, we equipped them with Radio Frequency Identification (RFID) tags. We also quantified one component of innate immune response by measuring encapsulation response, which has previously been used as a proxy of immunocompetence in leaf cutting ants^48^, honey bees^49^ and bumblebees^50^,^51^,^52^ where it correlates with parasite load and colony fitness^53^. We compared flight behaviour and encapsulation response of infected and non-infected bees within the same cohort and colony over a time frame of 4 days, which coincides with the successful establishment of a *N. apis* infection and the initial immune response of the host^54^.

## Results

### Course of *N. apis* infections

**Figure 1:**
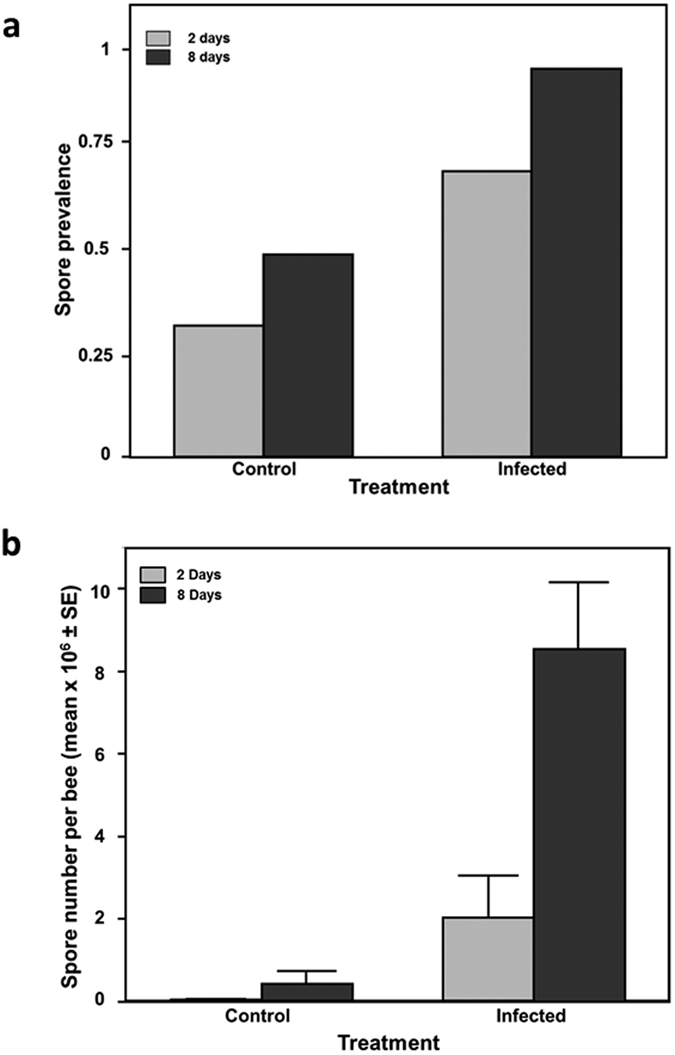
*Nosema* infections in honey bees 2 and 8 days after inoculation. Day 2 (grey bars), control (n = 37) and infected (n = 34) bees; day 8 (dark grey bars), control (n = 30) and infected (n = 25) bees. (**a**) Spores prevalence: (**b**) *Nosema* intensities, i.e. the total number of spores per bee.

A total of 126 bees became available for statistical analyses, which included 59 infected workers (19 from colony 1, 16 from colony 2 and 24 from colony 3) and 67 workers of the control treatment (17 from colony 1, 22 from colony 2 and 28 from colony 3). Parasite intensities were measured in 34 infected and 36 control bees 2 days after the inoculation treatment and in 25 infected and 30 control bees after 8 days from inoculation. We found that our inoculation treatment worked as expected, generating two distinct groups of workers with different *N. apis* infection levels. Parasite prevalence, measured as the proportion of infected bees, was significantly higher in workers fed with *N. apis* spores compared to workers from the control group (Wald χ^2^ = 27.588, p < 0.001), and higher on day 8 compared to day 2 (Wald χ^2^ = 15.679, p < 0.001). The difference in *Nosema* prevalence was already significantly different 2 days after infection (Wald χ^2^ = 10.592, p = 0.0011) and became further distinct 8 days after infection (Wald χ^2^ = 16.340, p < 0.001), indicating that infections continued to spread through our worker cohort over the 4 day period investigated (Fig. 1a). *Nosema* prevalence differed significantly among colonies (Wald χ^2^ = 24.457, p < 0.001) (Supplementary Figure S1a), but worker responses to infections were consistent among colonies as indicated by a non-significant treatment by colony interaction term (p = 0.809). Similarly, when analysing the intensity of the infections (the total number of spores in the gut of bees), we found that there was a significant effect of treatment (GLM; z-value = 2.744, p = 0.006) and days after inoculation (z-value = 3.312, p < 0.001), but no significant effect of colony (z-value = 0.153, p = 0.878) (Supplementary Figure S1b). All interaction terms were non significant (p > 0.05) (Fig. 1b).

### Flight behaviour

**Figure 2:**
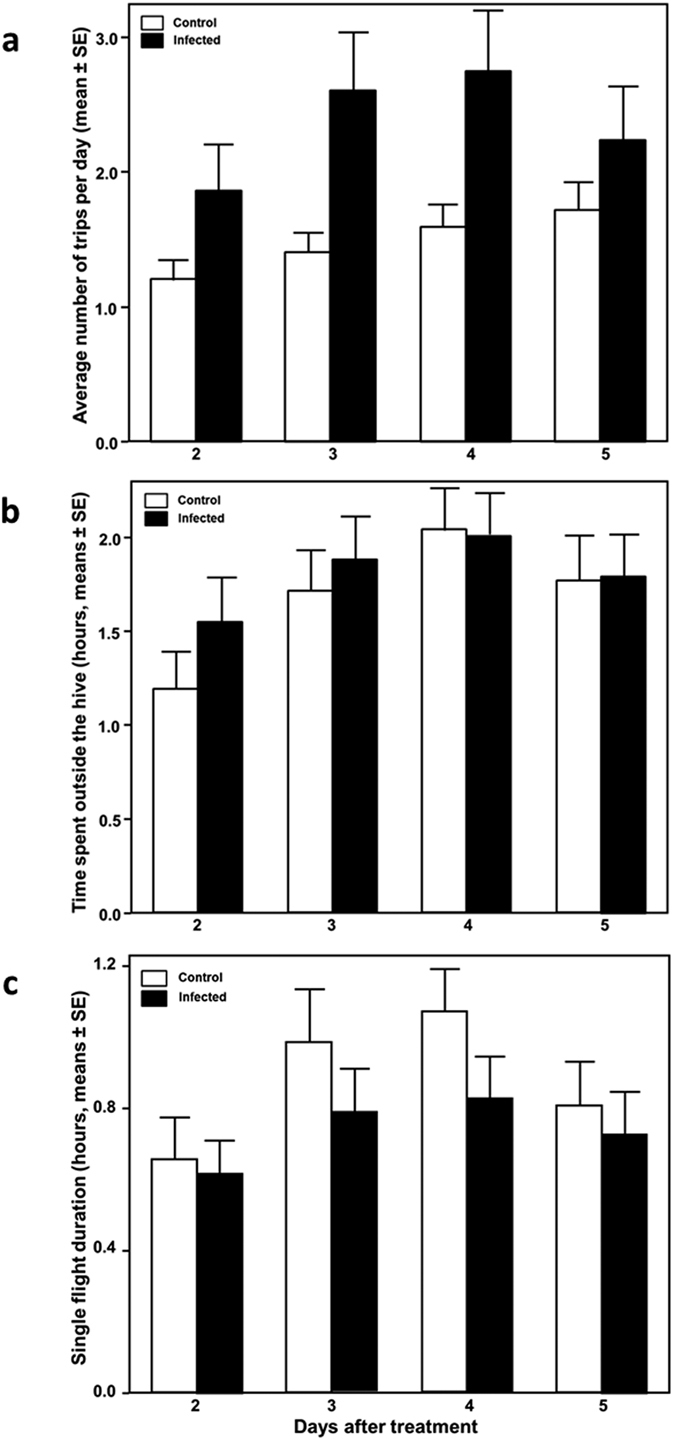
Flight parameters according to the day after treatment, in control (white bars, n = 90 bees; Colony 1, n = 25, Colony 2, n = 40 and Colony 3, n = 25) and infected (black bars, n = 80 bees; Colony 1, n = 21, Colony 2, n = 29 and Colony 3, n = 30) bees, recorded from day 2 to day 5 after treatment. Data shown are means and standard error of means. (**a**) Frequency of trips made to the outside of the hive, (**b**) Total time per day spent outside of the hive, (**c**) Single foraging flight duration.

**Figure 3:**
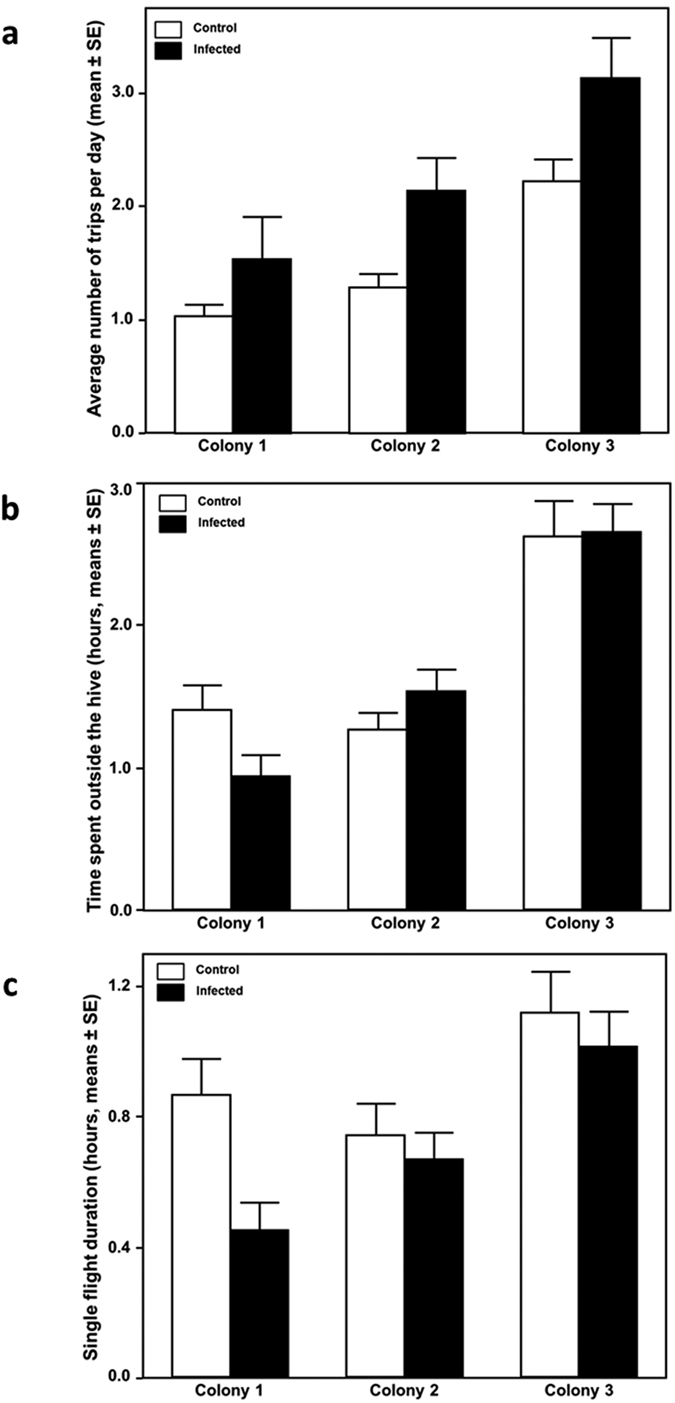
Flight parameters according to colony identity, in control (white bars, n = 90 bees; Colony 1, n = 25, Colony 2, n = 40 and Colony 3, n = 25) and infected (black bars, n = 80 bees; Colony 1, n = 21, Colony 2, n = 29 and Colony 3, n = 30) bees, recorded from day 2 to day 5 after treatment. Bars show means and standard error of means. (**a**) Frequency of trips per day for each colony in the experiment, (**b**) Time spent outside the colony for each colony, (**c**) Average duration of single flight for each colony.

Data on flight activities of workers became available for a total of 170 RFID equipped individuals (80 infected bees: 21 from colony 1, 29 from colony 2 and 30 from colony 3) and 90 uninfected bees: 25 from colony 1, 40 from colony 2 and 25 from colony 3) and complete reader recordings (see material and methods) became available for a total of 1274 individual flight trips. From this dataset we obtained information about flight frequency (number of times per day in which a bee made a complete flight, Figs 2a and 3a), overall flight duration (total time spent outside the hive per day, Figs 2b and 3b) and individual flight duration (duration of each individual flight, Figs 2c and 3c). We analysed how those parameters changed in control and infected bees in the days after infection (Fig. 2) and whether there were differences among our 3 experimental colonies (Fig. 3).

We found that workers infected with *N. apis* responded quickly to infections as they made significantly more trips compared to control bees (χ^2^ = 44.805, p < 0.001) (Fig. 2a). The frequency of trips changed significantly over the 4 days (χ^2^ = 18.472, p = 0.004) and differed between colonies (χ^2^ = 104.646, p < 0.001), the latter being driven by colony 3 where workers conducted more flights compared to the other colonies (Fig. 3a). All Interaction terms were non-significant (both p > 0.127).

Infected bees conducted shorter individual flights than non-infected ones (GLMM, F_1, 162_ = 4.120, p = 0.044, Fig. 2c), but flight durations increased with time, although this increase in trip length was not statistically significant (GLMM, F_3, 498_ = 2.531, p = 0.057). The length of flights differed between colonies (GLMM, F_2, 162_ = 6.797, p =  0.0015), because workers of colony 3 conducted significantly longer flights compared to workers of the other colonies (Fig. 3c). All interaction terms were non significant (p > 0.05).

Flight duration, measured as the total amount of time workers spent outside the hive did not differ between infected and non-infected bees (GLMM, F_1, 162_ = 0.077, p = 0.781, Fig. 2b) but bees increased their time spent foraging over the four days (GLMM, F_3, 498_ = 4.080, p = 0.007). Flight duration differed significantly among colonies (GLMM, F_2, 162_ = 21.526, p < 0.001) because workers of colony 3 spent significantly more time outside their hive compared to the other colonies (Fig. 3b). All interactions were non-significant (p > 0.05).

### Worker mortality

**Figure 4:**
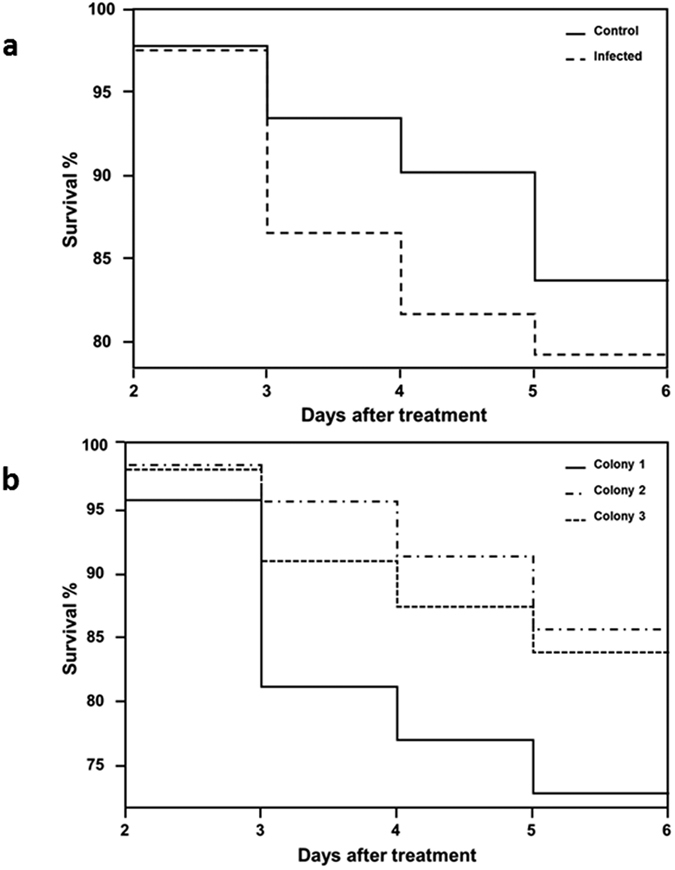
Survival analysis, from day 2 to day 5 after inoculation for control (n = 90) and infected (n = 80) honey bee workers. Values on y-axis are the percentage of foragers still alive at the given day point over the total initial bee population. (**a**) Effect of infection treatment on bee’s survival between treatments. Control bees (full line) and infected bees (dashed line). (**b**) Survival of workers for each of the three colonies showing Colony 1 as full line, Colony 2 as line and dots and Colony 3 as a dashed line.

Overall worker mortality was 22% over the 5 day period monitored, but worker survival did not differ among *N. apis* infected bees and the control group (Wilcoxon, χ^2^ = 0.820, p = 0.365) (Fig. 4a) or between the three colonies (overall comparison; Wilcoxon statistic: χ^2^ = 4.156, p = 0.125), although there was a trend for an overall lower survival of workers from colony 1 compared to colonies 2 and 3 (χ^2^ = 3.684, p = 0.055) (Fig. 4b). As a consequence, although we found a substantial number of workers not to survive until the end of the experiment, this must have been caused by factors other than *N. apis* infections.

### Encapsulation response

**Figure 5:**
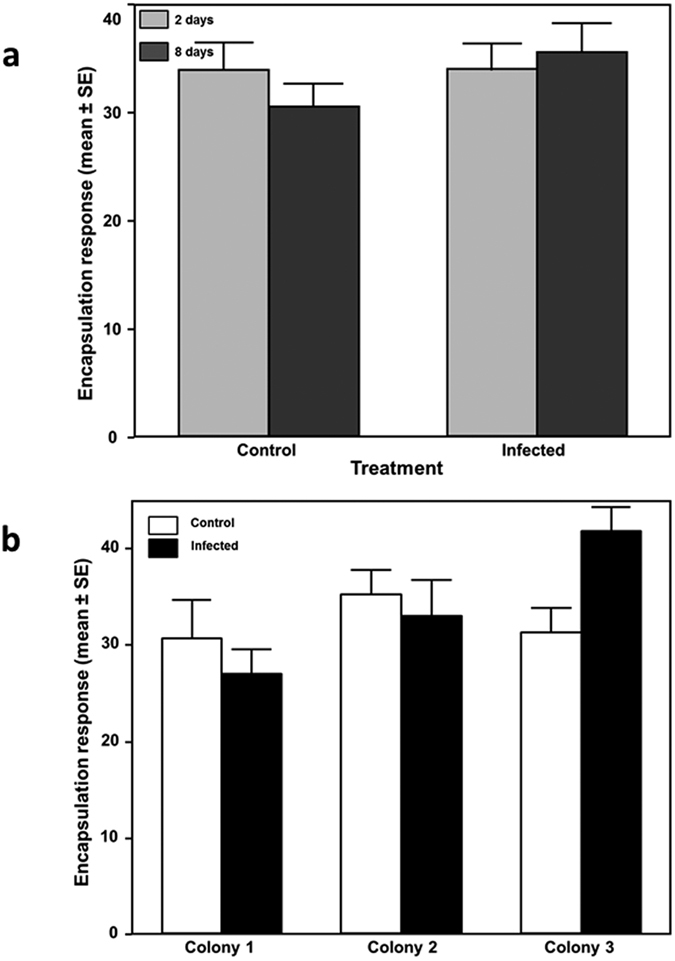
Encapsulation response assay results. (**a**) Effect of infection treatment per colony. Control bees (white bars): Colony 1 (n = 17), Colony 2 (n = 22), Colony 3 (n = 28), Infected bees (black bars): Colony 1 (n = 19), Colony 2 (n = 16), Colony 3 (n = 24). (**b**) Control vs infected bees on day 2 (grey bars), control (n = 36) and infected (n = 34) bees and day 8 (dark grey bars), control (n = 30) and infected (n = 25) bees.

Encapsulation response became available for the same 126 workers we used to quantify infection intensities (see above), but did not differ significantly between infected and non-infected bees (F_1, 119_ = 0.328, p = 0.568), or time after inoculation (F_1, 119_ = 0.328, p = 0.568) (Fig. 5a). A significant treatment by colony interaction term indicated that bees responded differently to infections depending on their colony of origin (F_2, 119_ = 3.743, p = 0.026) (Fig. 5b). Encapsulation response of workers was not affected by infection for colonies 1 and 2, but encapsulation response was significantly higher in infected workers in colony 3 (GLMM, χ^2^ = 7.742, p < 0.001, Fig. 5b).

## Discussion

We infected honey bee workers at the start of their foraging activity with a widespread fungal pathogen, which is known to trigger a response^55^ of an immune system able to recognise and kill the parasite. Our experimental protocol nevertheless triggered successful infections in spore fed workers that proliferated quickly in all animals investigated. Infection intensities increased more than 44-fold in workers between day 2 and 8 (see Fig. 1b, dark bars) but as expected from earlier work^54^,^56^, did not cause any significant increase in mortality over the time period investigated. Workers from the control treatment remained largely non-infected (Fig. 1), which implies that the infections we report throughout our experiment were primarily triggered by our initial experimental procedure rather than by workers picking up infections within or outside the hive.

We found that workers responded remarkably fast to infections as alterations in their flight activities were already observed at the first day of data collection, two days post infection (Fig. 2). Parasitized workers performed significantly shorter trips and increased the number of trips per day (Fig. 2), which could indicate that infected bees reduced their foraging range if flight duration correlates with foraging distance. This could be an adaptive response to avoid energetically costly long distance flights in order to exploit food sources closer to the colony. However, future research is now needed to test this idea, for example by monitoring foraging behaviour of honey bee workers using artificial feeding stations placed at different distances from the colony.

*Nosema* infections did increase worker encapsulation response although this was only observed in one out of the three colonies used for our experiments, indicating the presence of colony variation in immune responses against *Nosema*. A recent study that quantified proteomic changes in immune proteins within the seminal fluid of honey bee found all major insect immune pathways to be present and *N. apis* infections triggering abundance changes in immune proteins from several different pathways that were either up or down regulated^55^. Our finding of the presence of colony based variation in encapsulation responses after *N. apis* infections therefore ask for future investigations to quantify the regulation of those key genes identified earlier within these pathways and to test whether honey bees have multiple physiological ways to respond to *N. apis* infections. Such possible redundancy in immunity was already indicated through earlier work that found that the killing of *N. apis* spores can be triggered by at least two different molecules^39^.

Following these previous findings of complex immune responses in bees following an infection, we here provide field based evidence that they impact other life history traits, and our findings are therefore consistent with the idea for the presence of trade-offs between immunity and foraging behaviour in workers. The importance of energy availability for foraging duration and frequency has already been confirmed through previous research in ants and in bees^57^,^58^. For example, honey bee workers fed with a non-nutritious sugar solution reduce their overall activity but increase their foraging frequency to compensate for energetic shortfalls^57^. Furthermore, Mayack *et al*.^50^ found that the energetic state of honey bee workers is a more accurate determinant of their foraging behaviour than food and energy availability in the colony. Our findings support this idea, because our experimental setup tested infected and non-infected worker cohorts within the same colony and under identical colony conditions such as food availability. However, parasites inducing behavioural changes in foragers altering niche usage could have major effects on the colony level. Because honey bee colonies are essentially sessile organisms, a reduction in foraging range could increase their susceptibility to ecological stress, such as local food depletion or shortage. Further research is therefore required to understand how the alterations we quantified in foraging behaviour on the individual level translate into performance and fitness changes on the colony level.

Alternatively to the idea that the shorter flight trips we observed in infected individuals resulted from a reduction in foraging distance, infected bees might have left colonies more frequently to defecate. Infections of *N. apis* are indeed known to cause dysentery^30^,^33^. However, we monitored the bees with RFID tags over a relatively short period of time and at the early onset of infections and therefore during a time span when dysentery is normally not observed. Indeed, we found no faecal smears at the entrances of our colonies, which are the typical indicative signs of high *Nosema* infections in honey bees. Furthermore, we restricted our statistical analyses to trips longer than 60 seconds, because dysenteric bees are known to defecate immediately outside the colony and any such short trips would have been excluded from our final analyses.

We infected workers at an age that corresponded with the onset of their foraging activities, and quantified the effect of infection on foraging behaviour. Our experimental setup differed substantially from two previous studies quantifying the impact of *Nosema* on foraging where bees were infected immediately after hatching to study long-term effects of the parasite in chronically infected bees^16^,^59^. These earlier studied reported no effect of *Nosema* infections on foraging trip number and duration^59^ but bees workers significantly increased flight duration^16^. Our finding that foraging frequency and duration is reduced in newly infected workers therefore shows that the timing and progression of parasite infections are important key factors to consider when quantifying their effects on workers and colonies. Future research is therefore required to unravel those factors and conditions that trigger colonies to eventually collapse, which would have substantial value for the apiarian industry.

We found significant colony effects for a number of variables investigated and we have already pointed out that such variation could be key to unravel the sophisticated molecular interactions between parasite and its host. The variation detected was mainly driven by colony 3 where workers not only had significantly higher encapsulation responses compared to bees from the other colonies but also spent more time flying irrespective of whether they were infected or not. We kept all colonies under the same conditions and at the same location, and equalised their size and make up prior to the experiment. Genetically unrelated queens headed our experimental colonies, implying that genetic or non-genetic maternal effects, rather than environmental factors may have caused the observed colony differences detected in our data. Our findings are in line with previous studies reporting significant colony effects in bees responding to immune challenges including encapsulation response^40^,^49^,^60^ and in some cases they were linked to colony genetics^19^. Quantitative genetic studies could offer unique opportunities for future honey bee breeding towards managed stock with increased levels of disease tolerance.

## Materials and Methods

### Worker breeding and *Nosema apis* infection

All honey bees used in our experiments were kept in an apiary at the University of Western Australia between October and December 2013. We used three colonies kept in the same location and headed by unrelated queens that were all allowed to mate freely, and provided each of them with one frame of empty worker comb. We recollected combs with capped brood 20 days later and shortly before the eclosion of workers, moved them to an incubator at 32 °C and 55% humidity. We pooled hatching workers according to their colony of origin for up to 2 days and marked them on their thorax with different colours using a non-metallic paint marker. We marked at least 1000 workers per colony and glued a Radio Frequency Identification (RFID) tag on the thorax of a random subset of 200 bees per colony. All bees were afterwards released back into their maternal colony.

We recaptured marked bees from each colony on day 18 and 19 after eclosion and starved them for three hours in plastic boxes in an incubator at 32 °C and 55% humidity. Prior to the inoculation treatment, we chilled bees in a freezer at −20 °C for 7 minutes for easier handling. Previous research confirmed that these starvation and freezing procedures do not result in significant mortality^61^,^62^. Each bee was randomly allocated to one of two treatments (infected or control), and painted with an additional colour code to identify the treatment group and colony of each individual. Recaptured bees that carried a RFID tag were furthermore identified using a pen reader (ilD^®^ PENmini USB 7.0, 13.56 MHz). We then fed bees with either 2 μl of a 50% (w/v) sucrose solution (control) or 2 μl of a 50% sucrose solution containing 20,000 *Nosema apis* spores (infected). Spores used for inoculations originated from several colonies and were collected prior to the experiment using established protocols^63^. In brief, we collected 100 bees from a total of 4 colonies, freeze-killed them at −20 °C and macerated their abdomens with a mortar and pestle. We then filtered the extract with Whatman paper and centrifuged the sample at 20,000 g for 15 min. The spore containing pellet was dissolved in distilled water, layered onto 100% Percoll, and centrifuged at 20,000 g for 60 minutes at 4 °C. This procedure was repeated four times. Final spore concentration was determined using an Improved Neubauer haemocytometer and spores were kept at −80 °C prior to any further experiments. These procedures do not affect spore viability^63^.

We transferred bees to individual cages after the inoculation procedure and placed them in their maternal hives from where they were released the following morning. We allowed bees to recover from the experimental procedures for 24 hours before starting to record their flight activities. From a total of 600 bees that we initially tagged with RFIDs, a total of 201 tagged bees were recaptured and 170 bees finally provided complete flight trip data for statistical analyses, which included 90 control and 80 infected bees. We recaptured an additional 15 infected and 15 control bees per colony, 2 and 8 days after the inoculation and quantified both their immune response and *Nosema* spore loads as detailed below.

### Encapsulation response

To quantify the immune response of workers infected with *N. apis*, we used a previously established method to quantify immune responses in insects^48^,^51^,^64^,^65^ known as the encapsulation response. In short, a small piece of nylon is inserted into the insect’s haemocoel, where it is recognised by the insect’s immune system. Haemocytes form a melanised layer around the nylon, which can be quantified as a change in grey value of the implant^51^,^66^. To do this we anesthetized worker bees with CO_2_ for 2 minutes and placed them in a plastic holder mounted on modified artificial insemination equipment (Schley, Germany). Using injection needles we pierced a small hole into the intersegmental membrane between the second and third sternite and inserted a small piece of nylon (2 × 0.5 mm) into the bee’s haemocoel. Bees were allowed to recover and were afterwards kept in an incubator accompanied by bees of the same treatment and some sister workers. After 24 hours all bees were anaesthetised with CO_2_ and dissected to retrieve the implant which was mounted on a slide and embedded in Eukitt medium (Eukitt^®^, Fluka). Digital pictures of implants were taken using a digital camera (Canon EOS D30) mounted on a dissecting stereoscope (Leica MZ7.5, Leica). To quantify the degree of melanisation we used ImageJ software (version 1.45s) and measured the grey value of each implant from which we subtracted a grey value measure taken of the background. All measurements were performed with the experimenter being blind to treatment. All workers were afterwards frozen at −20° before further dissections to perform *Nosema* spore counts as described below.

### *Nosema* infection intensity and prevalence

We used a previously established protocol to quantify the intensity of *N. apis* infections^59^,^67^. Briefly, bees were thawed and their mid guts dissected and transferred to individual Eppendorf tubes containing 1 ml of deionized water. The gut tissue was gently macerated using a small pestle and vortexed for 2 minutes and a 7 μl subsample was loaded onto an improved Neubauer haemocytometer to count the number of spores in five squares using a light microscope (Leica DM 1000) at 400x magnification. Final spore concentrations were calculated by multiplying the total number of spores counted by a factor of 50,000^63^.

### Measuring flight behaviour of RFID tagged bees

**Figure 6:**
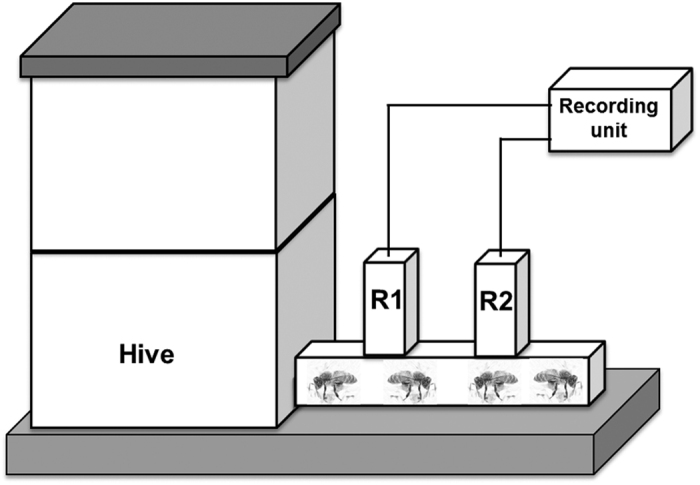
RFID readers setup on each colony, showing the set-up of the two readers (indicated as ‘R1’ and ‘R2’) relative to the hive entrance. the setup ensured that only a single bee could pass through a reader at a time, triggering a recording from each reader, and allowing to discriminate between flights outbound (read as R1–R2) or inbound (R2–R1).

We used a RFID setup previously used to monitor honey bee behaviour^59^,^68^,^69^, that allowed us to monitor bee movements in and out of hives^59^. To do this we modified the hive entrance forcing individual bees leaving and returning to their colony to pass through a set of two RFID tag readers (iID^®^ MAJA module 4.1 readers). The two readers were mounted on a wooden tube 200 mm long and placed in front of each of the 3 experimental hives (Fig. 6). The gap between the reader sensors and the floor was kept at 5–7 mm and was therefore of sufficient size for individual bees to pass through. This setup allowed us to identify a complete foraging trip by a sequence of recordings from the two readers, starting with a worker leaving the hive triggering the reader closer to the hive (inner) before the reader closer to the exit (outer), while returning bees triggering the outer before the inner reader. A complete foraging trip was therefore defined by 4 reader recordings in a defined sequence (in – out - out – in). All RFID equipment and software was purchased from Microsensys GmbH (Erfurt, Germany, www.microsensys.de) including RFID tags with individual bar codes (mic3^®^-TAG 64-bit RO). All raw data recoded by the readers were collected in XML format on an SD memory card in the database box (ilD^®^ HOST type MAJA 4.1) from where they were downloaded to a PC computer and assembled in a MySQL database. Here, we filtered the data as described previously^59^ before it was exported into Excel for statistical analysis. In short we collapsed multiple readings resulting in only a single event entry per tag for a given time. We also deleted all recordings that did not cover a complete sequence of reader recordings (in – out - out – in) and all readings occurring before sunrise and after sunset. As bees sometimes left the colony for very short periods of time we applied a threshold of 60 seconds to define a complete flight trip, a threshold applied in previous studies^59^,^68^,^69^. The RFID data collected allowed us to quantify the number of flights per day and the total daily flight duration per bee. To determine worker mortality, we used last date of recorded flight activity per bee and continued to monitor flight activity for an additional 6 days after the end of the experiments to confirm that bees we classified as having died during the 5-day time frame did not trigger further recordings.

### Statistical analyses

Statistical analyses were performed using JMP, version 11.0.0 (SAS Institute, USA) and using ‘R’ software version 3.1.2^70^. For all models we tested for the effect of treatment, day and colony as well as their interactions (treatment by day and treatment by colony). Details on the model are given for each variable tested; model assumptions were checked by visual inspection of residual plots. To analyse the prevalence of infection (proportion of infected bees) we used Generalized Linear Models with binomial error distribution and logit link because we found no indication that our raw data were overdispersed (overdispersion factor = 1.085). To statistically analyse *N. apis* intensities, we used a model with negative binomial error distribution and log link function (using ‘pscl’ package) in order to account for the large number of zero values in our dataset that originated from the non-infected bees in the control treatment^71^. The frequency of flights was analysed using a Generalized Linear Mixed Model (or GLMM) with a Poisson distribution. We included bee identity as a random factor to account for the non-independence of the data collected from the same individual. Flight duration and encapsulation response were analysed using linear mixed models. In the flight duration model, bee identity was included as a random factor.

Worker mortality was analysed on 170 individuals using life table analysis on JMP^72^. Survival distributions were estimated using the last flight time recordings of individuals up to 6 days post treatment. All workers that survived 5 days post treatment were included in the analysis as alive on day 6. The life table model assumes that the missing rate (probability of death at any given time point for an individual still alive) depends on a common baseline mortality, as well as the covariate effects of colony and treatment. Wilcoxon statistics were employed to investigate differences in mortality rate between variables.

## Additional Information

**How to cite this article**: Dosselli, R. *et al*. Flight behaviour of honey bee (*Apis mellifera*) workers is altered by initial infections of the fungal parasite *Nosema apis*. *Sci. Rep*. **6**, 36649; doi: 10.1038/srep36649 (2016).

**Publisher’s note:** Springer Nature remains neutral with regard to jurisdictional claims in published maps and institutional affiliations.

## Supplementary Material

Supplementary Information
