# Supplementary material for: Flight behaviour of honey bee (Apis mellifera) workers is altered by initial infections of the fungal parasite Nosema apis
Source: Sci Rep. 2016 Nov 9;6:36649. doi: 10.1038/srep36649 (PMC5101476; doi:10.1038/srep36649)
Supplement: Supplementary Information [file srep36649-s1.pdf]

Flight behaviour of honey bee *(Apis mellifera)* workers is altered by initial infections of the fungal parasite *Nosema apis*

Ryan Dosselli, Julia Grassl, Andrew Carson, Leigh W. Simmons & Boris Baer

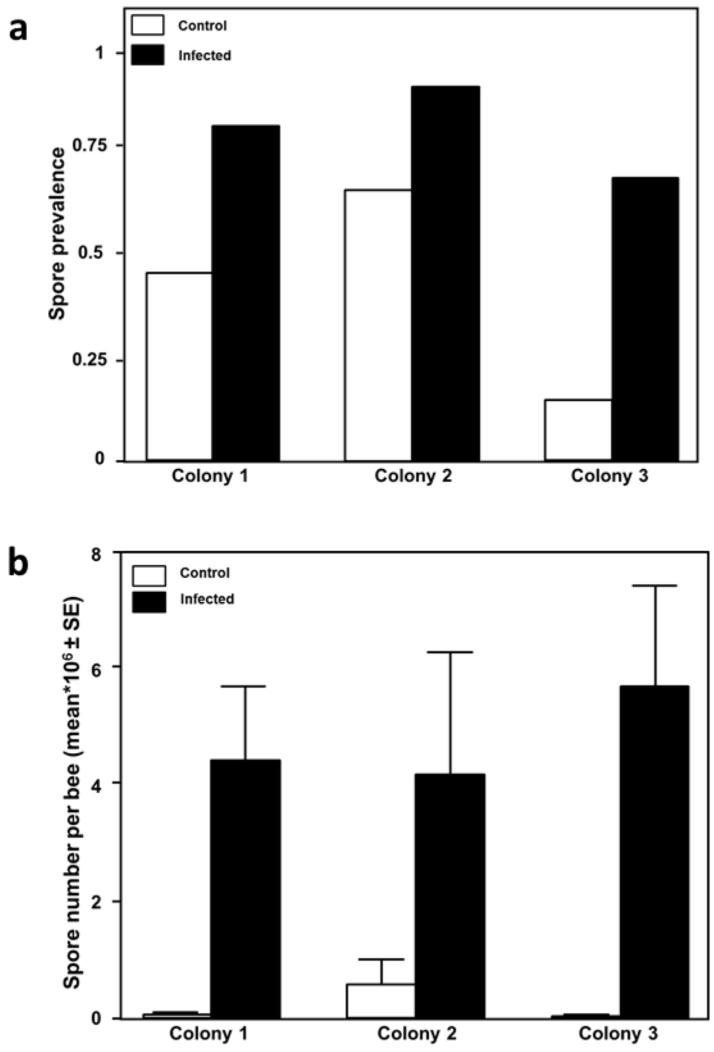

Figure S1. *Nosema* infections effect per colony. Control bees (white bars): Colony 1 (n=17), Colony 2 (n=22), Colony 3 (n=28), Infected bees (black bars): Colony 1 (n=19), Colony 2 (n=16), Colony 3 (n=24). (**a**) Spores prevalence; (**b**) *Nosema* intensities, i.e. the total number of spores per bee.
